# Semisupervised Clustering by Iterative Partition and Regression with Neuroscience Applications

**DOI:** 10.1155/2016/4037380

**Published:** 2016-04-26

**Authors:** Guoqi Qian, Yuehua Wu, Davide Ferrari, Puxue Qiao, Frédéric Hollande

**Affiliations:** ^1^School of Mathematics and Statistics, University of Melbourne, Parkville, VIC 3010, Australia; ^2^Department of Mathematics and Statistics, York University, Toronto, ON, Canada M3J 1P3; ^3^Department of Pathology, University of Melbourne, Parkville, VIC 3010, Australia

## Abstract

Regression clustering is a mixture of unsupervised and supervised statistical learning and data mining method which is found in a wide range of applications including artificial intelligence and neuroscience. It performs unsupervised learning when it clusters the data according to their respective unobserved regression hyperplanes. The method also performs supervised learning when it fits regression hyperplanes to the corresponding data clusters. Applying regression clustering in practice requires means of determining the underlying number of clusters in the data, finding the cluster label of each data point, and estimating the regression coefficients of the model. In this paper, we review the estimation and selection issues in regression clustering with regard to the least squares and robust statistical methods. We also provide a model selection based technique to determine the number of regression clusters underlying the data. We further develop a computing procedure for regression clustering estimation and selection. Finally, simulation studies are presented for assessing the procedure, together with analyzing a real data set on RGB cell marking in neuroscience to illustrate and interpret the method.

## 1. Introduction

Regression and clustering are probably two of the most important statistical data mining methods used in practice including artificial intelligence and neuroscience. However, regression clustering, a data mining method integrating the two, has rarely been studied as a single entity despite its great potential for practical use. It is, thus, the intention of this paper to focus on statistical estimation, selection, and computing of regression clustering. In this section, we briefly review cluster analysis but not the familiar regression analysis and then introduce the regression clustering problem.

*(1) Cluster Analysis*. Cluster analysis is an important unsupervised statistical learning and data mining technique for clustering homogeneous observations from data. Its main objective is to divide a collection of data points, often of multivariate nature, into subsets or “clusters” such that observations within one cluster are more “similar” (homogeneous) to each other than to observations in different clusters. Cluster analysis is usually used in situations where clustering information is not observed on the data points and one wants to get this information from the data to explicitly group them.

Many approaches have been developed in cluster analysis, which in general fall into two categories: hierarchical and partitive. A hierarchical approach proceeds by either a sequence of “agglomerative” stages or a sequence of “divisive” ones. At each agglomerative stage, clusters are produced by merging or retaining the clusters produced at the immediate previous stage, where clusters at the initial stage may simply be taken to be those individual data points. Contrarily, at each divisive stage, clusters are produced by splitting or retaining the clusters produced at the immediate previous stage, where one may assume a single cluster containing all the data points at the initial stage. The key feature of a hierarchical approach is that clusters obtained at one stage are derived from those in the immediate previous stage. On the other hand, partitive approaches refer to those nonhierarchical ones which may be further classified according to other features of clustering.

The outcome of a hierarchical clustering is often represented by a graph called dendrogram in which each stage of merging or splitting is determined by optimizing some similarity or dissimilarity criterion. A significant drawback of hierarchical clustering methods is that the divisions or fusions, once made, are irrevocable. That is, when an agglomerative algorithm has joined two objects into one cluster, they cannot subsequently be separated, and when a divisive algorithm has made an unwanted split, the objects involved can no longer be recombined into one cluster. Kaufman and Rousseeuw [1] comment on this as follows: “A hierarchical method suffers from the defect that it can never repair what was done in previous steps.”

In contrast, a partitive clustering constructs a fixed number of clusters often by an iterative procedure. It imposes two requirements in the procedure: (i) each cluster must contain at least one object and (ii) each object must belong to exactly one cluster. In addition, the number of clusters constructed stays fixed during the iterations and an initial partition is required to start the iteration. At each iteration, a tentative partition is constructed by relocating the data points to optimize a conditional criterion. This procedure continues until certain convergence or stability of partition occurs. Commonly used partitive clustering approaches include those *k*-means type of methods: *k*-means, *k*-modes, *k*-medians, and *k*-medoids [2, 3]. New developments in this regard can be found in Hastie et al. [4, section  14.3] and Clarke et al. [5, Chapter  8], for example.

**Figure 1:**
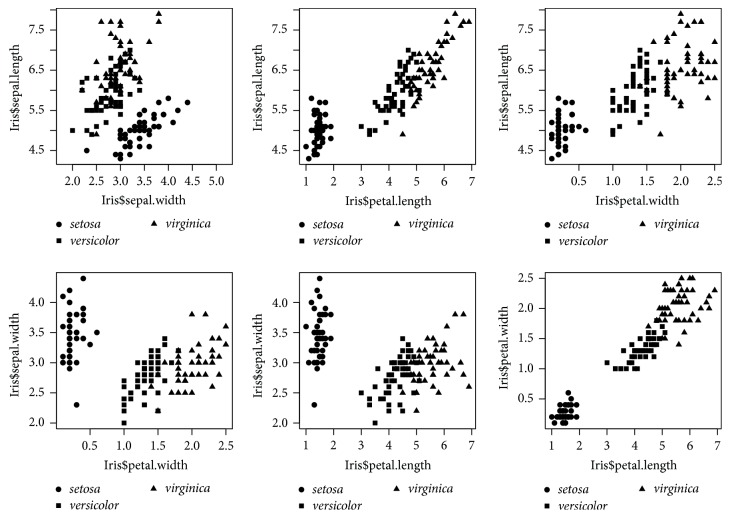
Pairwise scatterplots for the* Iris* data.

We present an example here to illustrate the use of *k*-means method for clustering. The example uses the well-known* Iris* data from Anderson [6] which was analyzed by Fisher [7] and many others. The data give the measurements in centimeters of the variables sepal length and width and petal length and width, respectively, for 50 flowers from each of the 3 species of* Iris*:* setosa*,* versicolor*, and* virginica*. The data which can be retrieved from statistics package R [8] are displayed in Figure 1, where we see the data of sepal length and width and petal length and width distributed in clusters. So we use the *k*-means algorithm of Hartigan and Wong [3] to find a partition of 3 clusters for the data and compare the partition with the species information given. The computing is done in R with a random initial partition determined by set.seed(123). The result is summarized in Table 1, from which we see a perfect match between cluster 1 and species* setosa* and some mismatch between clusters 2 and 3 and species* versicolor* and* virginica*.

*(2) Regression Clustering*. In this paper, we will focus on regression clustering, a data mining method which iteratively clusters data into clusters according to the available regression pattern and then updates the regression in each cluster simultaneously until equilibrium is attained. It is commonly known that regression is for studying the relationship between a dependent variable and a set of explanatory variables which have observations on a sample of objects. If the samples come from different populations and the variable indexing the populations also has an effect on the dependent variable, the regression should be performed on individual populations separately through the corresponding subsamples observed, or by including the population effect in the model, in order to make valid or more reliable statistical inference. However, the population indexing variable sometimes is not observed or unobservable. In such situations, it is necessary to cluster the sample objects to conform to their respective populations as much as possible and then apply regression to each cluster. We refer to this procedure as regression clustering if our focus is clustering the data points or as cluster regression if it is studying the unobserved regression patterns in the data.

Before getting into details of regression clustering, we review various measures of similarity or dissimilarity used in general cluster analysis. Note that to identify possible clusters of observations in data it is essential to be able to measure how close or how far individual data objects are to/from each other. Current measures include the single linkage (nearest neighbour) and complete linkage (further neighbour) (cf. [1]) and the *k*-means. These are usually considered as descriptive since they do not involve any probability distribution and use only descriptive statistics as the measures of similarity or dissimilarity between observations. An obvious disadvantage of using a descriptive measure is one cannot make statistical inference on results of clustering; thus, one is not able to assess the variability involved in the results. To enable making statistical inference, probability distributions or models are postulated for the clusters of data, and it is deemed that data in the same cluster have the same probability distribution. Hence, the similarity or dissimilarity measures to be used are assigned a probability distribution, and the significance and variability of clustering can be readily derived. Probability model based approaches can be applied in both hierarchical and partitive types of clustering. We choose to use the probability model for partitive regression clustering here.

Note that there is no absolute boundary between descriptive and probability model based clustering methods. Some clustering methods were heuristically motivated, but later on, statisticians studied their performance from a probabilistic perspective. For instance, MacQueen [2] and Pollard [9] studied the asymptotic behaviour of *k*-means using a probability model based approach; Hartigan [10] and Wong [11] investigated the mathematical relationship between high-density clusters and the single-linkage clustering method.

Consider a finite set of *n* objects *𝒪* = {1,…, *n*} together with data **z**_1_ = (*y*_1_, **x**_1_′)′,…, **z**_*n*_ = (*y*_*n*_, **x**_*n*_′)′ ∈ *ℝ* ^*p*+1^ being the observations of these objects. The problem of regression clustering is to recover the latent partitioning Π = (*𝒞*_1_,…, *𝒞*_*k*_) of *𝒪* so that the relationship between (*y*_1_,…,*y*_*n*_)′ and (**x**_1_,…, **x**_*n*_) can be studied by regressions on *𝒞*_1_,…, *𝒞*_*k*_ separately. A probability model based clustering approach assumes that the observed data **z**_1_,…, **z**_*n*_ are a sample of respective random vectors **Z**_1_,…, **Z**_*n*_ that belong to a set of populations indexed by Π. Thus, those **Z**_*j*_ with *j* ∈ *𝒞*_*i*_, *i* = 1,…, *k*, have the same probability distribution. The specification of the probability distributions can be either parametric or nonparametric.

*(3) Organization of the Paper*. In Section 2 we provide a detailed formulation of regression clustering including modeling, parameter estimation, and partition determination. In Section 3 we present two procedures for estimating the number of clusters in cluster linear regression. In Section 4, a pointwise iterative assessing algorithm is developed for implementing the regression clustering procedures. A simulation study and an example are presented in Section 5. A real data example on RGB cell marking clustering is analyzed in Section 6. The paper ends with a Conclusion section.

## 2. Regression Clustering Model and Optimization

Regression clustering becomes very useful when one intends to recover or estimate the unobserved class-specific regression hyperplanes based on the sample data of dependent and explanatory variables. Note that the notion of hyperplane used here is a generic one, which means it does not necessarily pass through the origin in the space. It should be more correctly called an affine set. But we do not distinguish them in this paper.

For cluster regression or regression clustering problem, the data have the form (*y*_*j*_, **x**_*j*_′), *j* = 1,…, *n*, where **x**_*j*_ ∈ *ℝ* ^*p*^ is an explanatory column vector and *y*_*j*_ ∈ *ℝ* is a random dependent variable for the *j*th object. The probability distribution of **x**_*j*_ does not provide any information on regression hyperplanes; thus, our statistical inference will be made conditional on the observed **x**_*j*_. In other words, we can simply treat **x**_*j*_ as nonrandom. As in the general setting of probability model based cluster analysis, there are two different approaches for regression clustering. One is the random partition or soft partition approach in which each data point is assigned a nonzero probability to fall into any of the clusters or equivalently follows a mixture probability distribution. The discussion can be found in DeSarbo and Cron [12] and Quandt and Ramsey [13], among others. Another one is the fixed partition or hard partition approach in which each data point is assigned a cluster membership or label through certain optimization procedure, so a data point belongs to only one cluster. As discussed in Bock [14, 15] and Späth [16, 17], the probability distribution or classification likelihood function of a data point in a fixed partition approach of regression clustering, with an unknown partition Π = (*𝒞*_1_,…, *𝒞*_*k*_) of *𝒪*, is of the form

(1) Yj~f·;βi,σi~ϕxj′βi,σi,∀j∈Ci,  i=1,…,k,

where in many situations we can assume *ϕ*(**x**_*j*_′**β**_*i*_, *σ*_*i*_) to be a normal density with mean **x**_*j*_′**β**_*i*_ and variance *σ*_*i*_^2^. This is equivalent to describing the data by a group of linear models:

(2) yj=xj′βi+ej,ej~N0,σi2,  ∀j∈Ci,  i=1,…,k.

Since the partition Π is unknown and the number of possible such partitions depends on *n*, the model (2) is a nonparametric one. Actually, it can be proved that the total number of nondegenerate partitions of form Π is equal to the Stirling number of the second kind $S(n,k)=\left(1/k!\right)\sum_{j=0}^{k}(-1{)}^{k-j}\left(\begin{array}{c} k\\ j \end{array}\right)j^{n}$; confer Tomescu [18]. Also, the linear regression function in (2) can be extended to a nonlinear one including spline and local polynomial regression and so forth under this regression clustering setting. This extension will not be pursued in this paper. Further, the true distribution of *e*_*j*_ need not be the normal. Namely, we use *N*(0, *σ*_*i*_^2^) only as a “working” distribution for *e*_*j*_. Then the corresponding least squares or maximum likelihood approach becomes the quasi-likelihood one that still possesses many optimality properties (cf. chapter 9 of [19]). We resort to using a robust approach in this paper instead to deal with the violation of normality assumption.

Given the regression clustering model introduced above, we need to estimate the parameters (**β**_*i*_, *σ*_*i*_^2^)_*i*=1,…,*k*_ and find the best partition Π together with *k* for application. Optimal parameter estimation and partition can be achieved using the maximum likelihood principle, while finding the optimal *k* can be done based on an information criterion. The latter will be explained in next section. Now, we proceed to do parameter estimation and partition.

Under the fixed partition model (2), the log-likelihood function is given by

(3) log⁡Lnk,Π,β1,σ12,…,βk,σk2=−12∑i=1k∑j∈Cilog⁡2π+log⁡σi2+yj−βi′xj2σi2.

It is clear that the best estimates of the parameters and the partition should be those maximizing the log-likelihood (3) for given *k*. However, the number of possible partitions *S*(*n*, *k*) is astronomic even for moderate *n* and *k*; for example, *S*(20,3) = 580,606,446 and *S*(50,3) ≈ 1.2 × 10^23^. Therefore, it is almost impossible to find the global optimal partition by enumeration. Here, we propose an iterative estimation method to find local optimal estimates of (**β**_*i*_, *σ*_*i*_^2^)_*i*=1,…,*k*_ and Π for a given *k*. This method extends the exchange method of Späth [16, 17].

When fixing (**β**_*i*_, *σ*_*i*_^2^)_*i*=1,…,*k*_ at given estimates $({\hat{\beta }}_{i},{\hat{\sigma }}_{i}^{2}{)}_{i=1,\ldots ,k}$, (3) achieves the maximum if each data point *j* belongs to cluster

(4) $$\begin{array}{c} {\hat{C}}_{i}=\arg\underset{1\leq i\leq k}{\min}\left(\;\log{\hat{\sigma }}_{i}^{2}+\frac{{\left(y_{j}-{\hat{\beta }}_{i}^{\prime }x_{j}\right)}^{2}}{{\hat{\sigma }}_{i}^{2}}\;\right). \end{array}$$

At given ${\hat{𝒞}}_{i}$, *i* = 1,…, *k*, (3) is the sum of the usual log-likelihood functions for homogeneous linear regressions within clusters. Hence, it is maximized at the least squares estimates ${\hat{\beta }}_{i}$ obtained based on the data points within ${\hat{𝒞}}_{i}$, and

(5) σ^i2=∑j∈C^iyj−β^i′xj2n^i,where  n^i=C^i  is  the  size  of  C^i,  i=1,…,k.

Then, $\log{\hat{L}}_{n}$ is monotonically increased if the steps (4) and (5) are carried out alternately. This procedure leads to a local maximum in finitely many steps. It is expected to be a good approximation of the global maximum if an initial partition is properly chosen. In practice, we often assume that the variance parameters *σ*_*i*_^2^, *i* = 1,…, *k*, have a common value *σ* ^2^ and estimate *σ* ^2^ by a pooled estimator. This modification tends to return a more robust partition than otherwise.

Note that the work in this section so far can be extended to multivariate regression clustering without any theoretical difficulty. The essential difference between multivariate regression and multiple regression is that the former has a vector response variable while the latter has a univariate one. Hence, (3) to (5) and the relevant ones in the rest of the paper can be easily modified to incorporate the vector response variable, from which it is ready to perform multivariate regression clustering. We will not get into the technical details involved but will provide a real data example in Section 6 to perform multivariate regression clustering.

It is well-known that the least squares method is very sensitive to outliers and violation of the normality assumption in the data. Robust methods can be developed to overcome this vulnerability. Among them, procedures based on *M*-estimation are considered here. *M*-estimation can be regarded as a generalization of the maximum likelihood estimation. A particular one is the maximum likelihood estimation based on Huber's least favourable distribution, whose density function is the normal at around the origin and the exponential in the tails. Using Huber's *M*-estimation method, we can drop the assumption *e*_*j*_ ~ *N*(0, *σ*_*i*_^2^) in (2) and estimate **β**_*i*_ by minimizing $\sum_{j\in {\hat{𝒞}}_{i}}\rho_{c}(y_{j}-\beta_{i}^{\prime }x_{j})$ for given partition ${\hat{𝒞}}_{i},\;\;i=1,\ldots ,k$. Here, *ρ*_*c*_(·) is Huber's discrepancy function defined as

(6) $$\begin{array}{c} \rho_{c}\left(\;t\;\right)=\left\{\begin{array}{cc} \frac{1}{2}t^{2}, & \left|t\right|<c,\\ c\left|t\right|-\frac{1}{2}c^{2}, & \left|t\right|\geq c, \end{array}\right. \end{array}$$

where *c* is determined by the scale parameter in Huber's least favourable distribution. We find that assuming a constant scale parameter across all clusters tends to give better robust results, so we adopt this assumption in this paper. Now for given estimates ${\hat{\beta }}_{i},\;\;i=1,\ldots ,k$, each data point *j* is assigned or reassigned to cluster ${\hat{𝒞}}_{i}=\arg\min_{1\leq i\leq k}\rho_{c}(y_{j}-{\hat{\beta }}_{i}^{\prime }x_{j})$. At this point, it can be seen that, instead of $\log{\hat{L}}_{n}$, the function ∑_*i*=1_^*k*^∑_*j*∈*𝒞*_*i*__ *ρ*_*c*_(*y*_*j*_ − **β**_*i*_′**x**_*j*_) will be monotonically increased if the above two *M*-estimation steps are carried out alternately. This gives a robust counterpart of the likelihood-based local optimal estimation and selection introduced earlier in this section.

**Figure 2:**
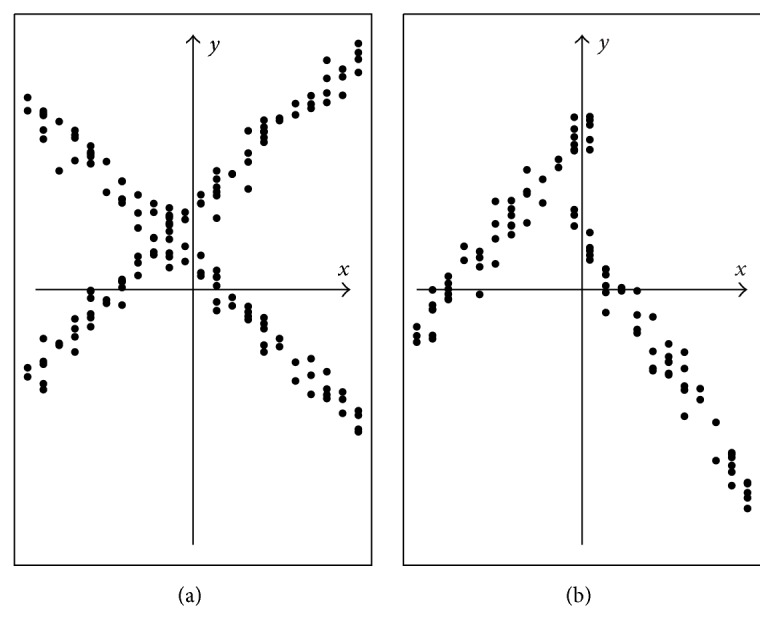
Assignment independence (a) versus assignment dependence (b).

To conclude this section, note that the fixed partition approach has a particular advantage over the random partition one in the context of regression clustering or cluster regression. As observed by Hennig [20], the mixture probability model involved in random partitioning presumes implicitly an* assignment independence* of each object to clusters with respect to the covariate vectors **x**_*j*_. That is, the clusters keep the same proportions {*π*_*i*_, *i* = 1,…, *k*} for every fixed covariate vector **x**_*j*_. In other words, the probability of a point (*y*_*j*_, **x**_*j*_′) to be generated by cluster *i* is independent of **x** and *j*. This is generally not true as shown in Figure 2, which is adapted from Hennig [20]. On the other hand, the fixed partition model (2) supposes that the cluster membership of each object or cluster labels are explicitly parameterized and are determined by the estimation of ${\hat{\beta }}_{i}$ and ${\hat{\sigma }}_{i}^{2}$ through the points (*y*_*j*_, **x**_*j*_′)  , *j* ∈ *𝒞*_*i*_. Hence, the fixed partition model does take care of the problem of possible* assignment dependence* between the *j*th object and the associated covariate **x**_*j*_. In principle, the random partition approach can be generalized to account for the assignment dependence, for example, by allowing {*π*_*i*_, *i* = 1,…, *k*} to depend on **x**_*j*_. But the resultant probability model will be much more difficult to be analyzed both algebraically and numerically; and no such study can be found in literature so far to our knowledge.

## 3. Estimating the Number of Clusters

The number of clusters to be used in regression clustering is normally unknown so it should also be estimated. In this section we provide two procedures for estimating the number of clusters, one based on least squares estimation and the other on robust *M*-estimation.

We use a more detailed notation *𝒪* ^(*n*)^ = {1,2,…, *n*} to denote the *n* data objects which have observations (*y*_1_, **x**_1_′),…, (*y*_*n*_, **x**_*n*_′) as described in previous sections. Recall that these *n* objects are assumed to be a random sample coming from a structured population, which consists of a fixed (but unknown) number, say *k*_0_, of subpopulations, each of which is characterized by a regression hyperplane with class-specific unknown parameters. Therefore, for the *n* observations from this population, there exists an underlying partition Π_*k*_0__^(*n*)^ = {*𝒪*_1_^(*n*)^,…, *𝒪*_*k*_0__^(*n*)^}, and by (2) each cluster *𝒪*_*i*_^(*n*)^≜{*i*_1_,…, *i*_*n*_*i*__}⊆*𝒪* ^(*n*)^ follows the regression model

(7) $$\begin{array}{c} y_{O_{i}}=X_{O_{i}}\beta_{0i}+e_{O_{i}},\;e_{O_{i}}\sim N\left(0,\sigma_{i}^{2}I_{n_{i}}\right), \end{array}$$

where **y**_*𝒪*_*i*__ = (*y*_*i*_1__,…,*y*_*i*_*n*_*i*___)′, *X*_*𝒪*_*i*__ = (**x**_*i*_1__,…,**x**_*i*_*n*_*i*___)′ is an *n*_*i*_ × *p* design matrix in the cluster *𝒪*_*i*_, **e**_*𝒪*_*i*__ is an *n*_*i*_-vector of random errors, *I*_*n*_*i*__ is an *n*_*i*_ × *n*_*i*_ identity matrix, and *n*_*i*_ = |*𝒪*_*i*_| for *i* = 1,…, *k*_0_. Here, (**β**_0*i*_′, *σ*_*i*_)′ ∈ *ℝ* ^*p*^ × *ℝ* ^+^, 1 ≤ *i* ≤ *k*_0_, are *k*_0_ unknown parameter vectors; and **β**_0*i*_, 1 ≤ *i* ≤ *k*_0_, are assumed to be distinct from one another. It is clear that *n* = *n*_1_ + ⋯+*n*_*k*_0__. In the following, we assume that *k*_0_ ≤ *K*, where *K* is a known positive integer. Note that in (7) we have suppressed the *n* in *𝒪*_*i*_^(*n*)^ for simplicity of presentation. Also note that the normality assumption for the random errors **e**_*𝒪*_*i*__, although reasonable in many situations, is just a “working” distribution and not really required for applying the least squares method.

In order to estimate *k*_0_, we fit a regression clustering model to the data for each *k* ≤ *K* using the methods developed in Section 2. A criterion function of *k* can be obtained from the cluster regression fitting. Then *k*_0_ is estimated as the minimizer of the criterion function. Shao and Wu [21] have used this idea to develop an information-based criterion for estimating *k*_0_. Let Π_*k*_^(*n*)^ = {*𝒞*_1_^(*n*)^,…, *𝒞*_*k*_^(*n*)^} be an arbitrary *k*-cluster partition of the *n* observations. Shao and Wu's information criterion is defined as

(8) $$\begin{array}{c} D_{n}\left(\;\Pi_{k}^{\left(n\right)}\;\right)=\overset{k}{\underset{i=1}{\sum }}{\left\|\;y_{C_{i}^{\left(n\right)}}-X_{C_{i}^{\left(n\right)}}{\hat{\beta }}_{i}\;\right\|}^{2}+q\left(\;k\;\right)A_{n}, \end{array}$$

where *q*(*k*) is a strictly increasing positive function of *k*, *A*_*n*_ is a sequence of positive constants, ${\hat{\beta }}_{i}$ are least squares estimators, and ‖·‖ is the Euclidean norm. Typically, *q*(*k*) = *kp* and *A*_*n*_ ∝ log⁡(*n*) or *A*_*n*_ ∝ log⁡log⁡(*n*) are chosen. Then ${\hat{k}}_{n}$, the estimate of *k*_0_, is the integer that minimizes this criterion, that is,

(9) $$\begin{array}{c} D_{n}\left(\;{\hat{k}}_{n}\;\right)=\underset{1\leq k\leq K}{\min}\;\underset{\Pi_{k}^{\left(n\right)}}{\min}D_{n}\left(\;\Pi_{k}^{\left(n\right)}\;\right). \end{array}$$

It can be seen that in (8) the first term is the sum of residual squares which measures the goodness of fit of the model and the second term is the penalty for overfitting. Moreover, the criterion (9) shows that one determines the optimal number of clusters and the corresponding partitioning simultaneously. We shall call (8) together with (9) Criterion LS-C in the sequel, which stands for clustering by the LS method.

Under some mild conditions, it is shown in Shao and Wu [21] that the proposed Criterion LS-C selects the true number of regression hyperplanes with probability one among all class-growing sequences of classifications, when the number of observations *n* from the population increases to infinity.

Concerning the robustness of regression clustering, one can use a robust criterion to estimate the underlying number of clusters *k*_0_, where we assume that each cluster *𝒪*_*i*_≜{*i*_1_,…, *i*_*n*_*i*__}⊆*𝒪* ^(*n*)^ is characterized by a linear model:

(10) $$\begin{array}{c} y_{j,O_{i}}=x_{j,O_{i}}^{\prime }\beta_{0i}+e_{j,O_{i}},\;j\in O_{i}, \end{array}$$

with the random error *e*_*j*,*𝒪*_*i*__ not following any specific distribution contrary to that in the linear model (7). In particular, Rao et al. [22] have developed the following robust information criterion function for estimating *k*_0_:

(11) $$\begin{array}{c} R_{n}\left(\;\Pi_{k}^{\left(n\right)}\;\right)=\overset{k}{\underset{s=1}{\sum }}\underset{j\in C_{s}}{\sum }\rho_{c}\left(\;y_{j,C_{s}}-x_{j,C_{s}}^{\prime }{\hat{\beta }}_{s}\;\right)+q\left(\;k\;\right)A_{n}, \end{array}$$

where *ρ*_*c*_ is Huber's discrepancy function and ${\hat{\beta }}_{s}$ are the *M*-estimators described in Section 2 or equivalently satisfying

(12) ∑j∈Csρcyj,Cs−xj,Cs′β^s=minβs⁡∑j∈Csρcyj,Cs−xj,Cs′βs.

It can be seen that similar to that in (8) the first term in (11) is a generalization of a minimum negative log-likelihood function derived from Huber's least favourable distribution, and the second term is the penalty for overfitting.

Using (11), the estimate ${\hat{k}}_{n}$ of the underlying number of clusters *k*_0_ is the one satisfying

(13) $$\begin{array}{c} R_{n}\left(\;{\hat{k}}_{n}\;\right)=\underset{1\leq k\leq K}{\min}\;\underset{\Pi_{k}^{\left(n\right)}}{\min}R_{n}\left(\;\Pi_{k}^{\left(n\right)}\;\right). \end{array}$$

We shall call (11) together with (13) Criterion RM-C, which stands for the clustering based on robust *M*-estimation. Similar to Criterion LS-C, Criterion RM-C implies that one determines the optimal number of clusters and the corresponding partitioning simultaneously.

In Rao et al. [22], it is shown that the true clustering and the associated regression hyperplanes are attained with probability 1 by RM-C when *n* increases to infinity and under certain mild conditions. In particular, normal distribution assumption is not required for the random errors in each regression cluster.

## 4. Pointwise Iterative Algorithms for Regression Clustering Estimation, Partition, and Selection

Computing algorithms can be written to implement the regression clustering methods described in Sections 2 and 3. Recall that in the methods we first estimate the optimal partition Π_*k*_ = {*𝒞*_1_,…, *𝒞*_*k*_} and the regression parameters simultaneously by minimizing certain within-cluster sum of residual squares sums (SRSS) or alike for each fixed *k*. The quantity to be minimized is equivalent to

(14) $$\begin{array}{c} \text{SRSS}\left(\;\Pi_{k},\beta_{1},\ldots ,\beta_{k}\;\right)=\overset{k}{\underset{i=1}{\sum }}{\left\|\;y_{C_{i}}-X_{C_{i}}\beta_{i}\;\right\|}^{2} \end{array}$$

for LS regression clustering or sum of robust residual squares sums (RRSS)

(15) $$\begin{array}{c} \text{RRSS}\left(\;\Pi_{k},\beta_{1},\ldots ,\beta_{k}\;\right)=\overset{k}{\underset{i=1}{\sum }}\overset{n_{i}}{\underset{j=1}{\sum }}\rho_{c}\left(\;y_{j,C_{i}}-x_{j,C_{i}}^{\prime }\beta_{i}\;\right) \end{array}$$

for an *M*-estimation based robust regression clustering. Only local minimization results can be guaranteed here. We process this local minimization for each candidate *k* and use Criterion LS-C or RM-C to determine the best *k*. The whole procedure can be accomplished according to the following algorithm:

(i) Label all the observations from 1 to *n* (order does not matter). Given an initial partition Π_*k*_ = {*𝒞*_1_,…, *𝒞*_*k*_} of *𝒪* = {1,…, *n*}, fit a regression model (or a robust regression model with a *ρ*_*c*_(·) function for RM-C criterion) in each of the *k* clusters and obtain the sum of the residual squares sums SRSS_0_ or RRSS_0_ for this partition. Let *i* = 0.
(ii) Set *i* = *i* + 1, and reset *i* = 1 if *i* > *n*. Identify *𝒞*_*j*_ such that *i* ∈ *𝒞*_*j*_. Then move *i* into *𝒞*_*h*_ with *h* = 1,…, *k* and *h* ≠ *j*, respectively. For each of these *k* − 1 relocations, refit the model by regression clustering (or robust regression clustering) and calculate the sum of the residual squares sums (or RRSS) accordingly. Denote the smallest one by SRSS_*h*_ or RRSS_*h*_. If SRSS_*h*_ < SRSS_0_ (or RRSS_*h*_ < RRSS_0_ in robust procedure), redefine *𝒞*_*j*_ = *𝒞*_*j*_ − {*i*} and *𝒞*_*h*_ = *𝒞*_*h*_ + {*i*}, and set SRSS_0_ = SRSS_*h*_ (or RRSS_0_ = RRSS_*h*_). Otherwise, return to the beginning of (ii).
(iii) Repeat (ii) until the objective function (14) or (15) does not decrease any further, which means that no observation relocation is necessary and the optimal clustering is achieved for this *k*.
(iv) Proceed with (i) to (iii) for each candidate *k* and use the Criterion LS-C or RM-C to find ${\hat{k}}_{n}$, the optimal number of clusters.

It is important to use a good initial partition of {1,…, *n*} in running steps (i) to (iii) so that the global minimum of (14) or (15), or its good approximation, can be achieved. We propose to generate the initial partition of a dataset using the following algorithm which we find works well in practice:

(I1) Consider the linear model
  (16) $$\begin{array}{c} y_{i}=x_{i}^{\prime }\beta +e_{i}. \end{array}$$
  Based on the whole dataset, one estimates **β** by a robust method, for example, least median regression or least trimmed squares method [23]. Note that a random seed is implicitly used in such robust methods.
(I2) Put into set *C*_1_ those data points whose distances to the regression hyperplane estimated in Step (I1) are less than a predetermined number, say *δ*. If |*C*_1_| and |*C*_1_^*c*^| are both larger than a predetermined integer, say *m*, set *ℓ* = 1 and go to the next step; otherwise, set *ℓ* = 0 and go to Step (I5). Here, *C*_1_^*c*^ is the complementary set of *C*_1_.
(I3) Based on the dataset ⋂_*i*=1_^*ℓ*^ *C*_*i*_^*c*^, one estimates **β** in (16) by the same robust method used in Step (I1).
(I4) Put into *C*_*ℓ*+1_ those points in ⋂_*i*=1_^*ℓ*^ *C*_*i*_^*c*^ whose distances to the regression hyperplane estimated in Step (I3) are less than *δ*. If |*C*_*ℓ*+1_| and |⋂_*i*=1_^*ℓ*+1^ *C*_*i*_^*c*^| are both larger than *m*, set *ℓ* = *ℓ* + 1 and repeat Step (I3); otherwise, go to Step (I5).
(I5) The initial partition is {*C*_1_,…, *C*_*ℓ*_, ⋂_*i*=1_^*ℓ*^ *C*_*i*_^*c*^} if *ℓ* > 1 or just the whole dataset itself if *ℓ* = 0.

One can adjust the values of *δ* and *m* either in advance or adaptively to get an initial partition of *k* clusters for any given *k*. For example, set *m* to the integer part of 0.5*n*/*k* and *δ* to the best value such that two clusters can be obtained in (I2).

The above initial partition algorithm gives essentially an iterated hierarchical binary clustering method, where each binary clustering is realized through resistant regression such as the least median regression. The resistant regression is robust, having high breakdown threshold; thus, although not being fully efficient, it is highly likely to produce a reasonable initial partition through its iterated executions.

The two algorithms consisting of Steps (I1) to (I5) and (i) to (iv) may be named IPARC to reflect the iterative pointwise assessing nature in regression clustering.

## 5. Example and Simulation Study

In this section, we first apply regression clustering to the* Iris* data and provide a brief guideline on when to use the method properly. We then present a simulation study to assess the finite sample performance of Criteria LS-C and RM-C.

### 5.1. The Iris Data Example

Recall the* Iris* data that we analyzed using the *k*-means method in Section 1. Now we want to use the regression relationship between sepal length and sepal width variables to partition the 150 observations of sepal length and width and petal length and width into 3 clusters. Statistics package R is used to implement our IPARC procedure, where we set *m* = 2*p* and *δ* = 0.05 or 0.2 and initial random seed being determined by set.seed(123456), and use only the least squares estimation in this example. The partition result and its comparison with the species information are summarized in Table 2. Comparing Tables 1 and 2, we see that the cluster information revealed by the cluster regression sepal.length ~ sepal.width is very much the same as that by the *k*-means and conforms with the species information.

When we use cluster regression sepal.length ~  sepal.width + petal.length + petal.width to partition the data into 3 clusters, we get a result summarized in Table 3 which is very different from Tables 1 and 2. This confirms that the cluster label information obtained from regression clustering has a different interpretation from that obtained from the *k*-means. The former tells us how differently regression performs across the clusters, while the latter tells us how distances among data observations themselves behave differently across the clusters. Fitting this regression clustering to the data gives SRSS = 2.901 and coefficients of determination of 0.972, 0.958, and 0.971, respectively, for the 3 regression hyperplanes. On the other hand, when we fit the same regression model to the 3 clusters determined by the *k*-means, we get SRSS = 12.699 and coefficients of determination of 0.575, 0.525, and 0.578. Similar results are obtained if the same regression model is fit to the 3 clusters determined by the species variable. Therefore, regression clustering method is fundamentally different from the general cluster analysis methods such as the *k*-means. One should use regression clustering if partitioning data to conform to the regression pattern is of interest.

### 5.2. Simulation Study

We use simulated data sets to assess the finite sample performance of Criteria LS-C and RM-C for regression clustering. Two factors will be considered for this simulation: number of clusters (2 or 3) and error distributions (*N*(0,1), *t*(3), or Cauchy(0,1)), so there are in total 6 cases of data to be considered, which are summarized in Table 4. There will be only one covariate involved in the regression clustering and the covariate is generated from *N*(0,1). The parameters used for each case are given in Table 5. Then, the fixed partition regression clustering model *y*_*ji*_ = **x**_*ji*_′**β**_0*i*_ + *e*_*ji*_, *j* = 1,…, *n*_*i*_, *i* = 1,…, *k*_0_, is applied to generate the response values *y*_*ji*_, where *e*_*ji*_ is a random number originating from *N*(0,1), *t*(3), or Cauchy(0,1), and the first element of **x**_*ji*_ is the constant 1 corresponding to the intercept term in the model.

**Figure 3:**
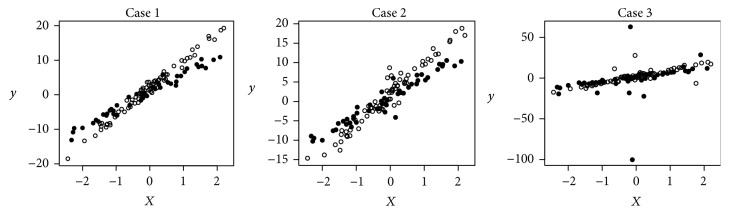
Simulated data with two clusters.

**Figure 4:**
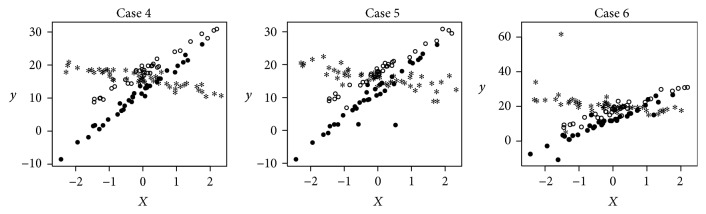
Simulated data with three clusters.

Figures 3 and 4 give us an intuition of what the data typically look like for Cases  1–6 with normal, *t*(3), or Cauchy errors. These figures show that the groupings of the linear patterns are visible with standard normal random errors and getting worse with *t*(3) random errors. The groupings are hard to see with Cauchy(0,1) random errors.

In this study, we set *q*(*k*) = *kp*, where *p* is the number of regression coefficients in the model and is a constant in our study; *k* is the unknown number of clusters that we are seeking. It is noted that in an information model selection criterion, a penalty function, which is *A*_*n*_ in (8) or (11), is usually chosen as *c*log⁡(*n*) or *c*log⁡log⁡(*n*) with a constant *c* > 0. In light of the fact that lim_*λ*→0_[(log⁡*n*)^*λ*^ − 1]/*λ* = log⁡log⁡*n*, we set *A*_*n*_ = [(log⁡*n*)^3^ − 1]/3.

The *ρ*_*c*_ function we employed for *M*-estimation is *ρ*_*c*_(*u*) = 0.5*u* ^2^ if |*u*| ≤ 1.345 and *ρ*_*c*_(*u*) = 1.345|*u*| − 0.5 × 1.345^2^ otherwise (Huber *ρ*_*c*_). In the following, when we state the simulation results, Criterion RM-C means *M*-estimation based regression clustering procedure with Huber's *ρ*_*c*_ exclusively.

For each of the six cases, we conduct 1000 simulations using Criteria LS-C and RM-C separately. To apply the algorithm IPARC, we set *δ* = 0.2 and *m* = 2*p*. The algorithm given in the previous section is then used to estimate the number of clusters in cluster linear regression. In Tables 6 and 7, we summarize the results from the simulation study, where each number represents the relative frequencies of selecting the possible numbers of clusters *k* out of 1000 replications.

It is clear that Criterion LS-C performs almost perfectly in Cases  1 and 4 since the errors are standard normal distributed. However, when there exist outliers in the data set or the normality of the data is violated, Criterion LS-C performs poorly. On the contrary, as shown in Tables 6 and 7, Criterion RM-C does as nearly perfect a job as Criterion LS-C in Cases  1 and 4; at the same time, neither outliers nor abnormality has much effect on its ability to detect the underlying true number of regression hyperplanes in the data.

In addition to the robustness shown above in selecting the number of clusters, the procedure of the *M*-estimation based regression clustering is also robust in partitioning the data.  Table 8 presents the estimation of the regression parameters by applying LS-C and RM-C to the data shown in Figures 3 and 4. From the table, it can be seen that when the errors are *t*(3) or Cauchy(0,1) distributed, the LS regression clustering method is not able to capture the underlying groupings, while the *M*-estimation based regression clustering method detects the true linear patterns in the data, in spite of the abnormality in the data.

## 6. Analysis of RGB Cell Tracking Data

**Figure 5:**
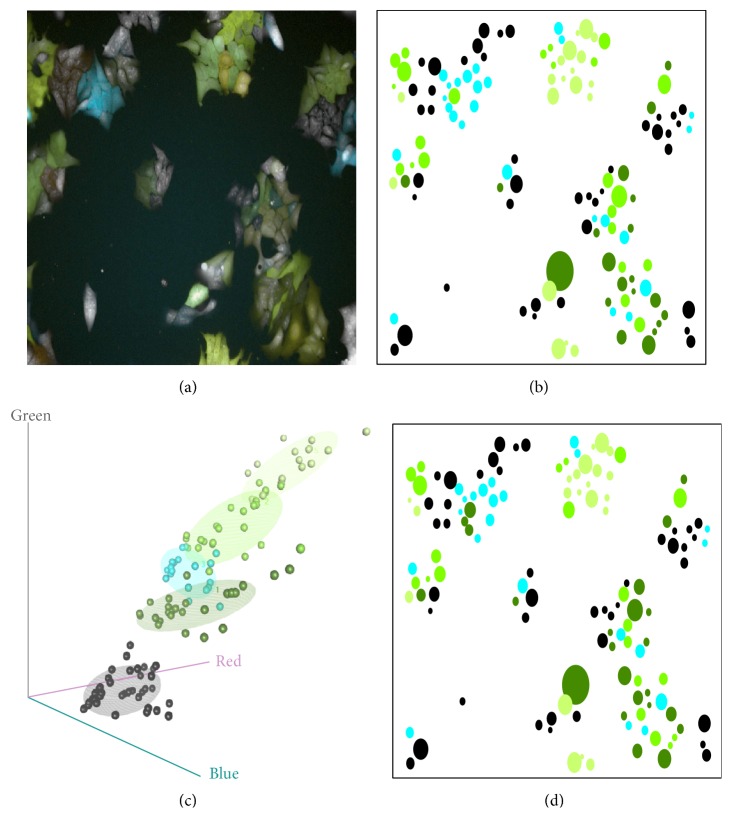
(a) Raw spatial data on 128 colorectal cancer cells imaged on a high-content microscope imager (Operetta, Perkin Elmer). (b) Spatial distribution of the 128 cells represented by the colored circles. The circles have 5 colors representing 5 clusters which resulted from LS multivariate regression clustering minimizing (14). The size of each circle estimates the area of the corresponding cell. The clustering uses RGB intensities as the response vector and cell area as the predictor. (c) 3D-scatterplot of the clustered RGB intensities of the 128 cells. Colors of the points show the same 5 clusters shown in (b). (d) Spatial distribution of the 128 cells, with the colored circles showing the 5 clusters given by the LS multivariate regression clustering not including any predictor.

Recently, a new technique called RGB marking has been introduced to facilitate the identification of individual cell clones in both in vivo and in vitro experiments [24]. RGB marking introduces three lentiviral vectors in individual cells encoding the basic colors red, green, and blue. Raw image data representing 128 colorectal cancer cells are shown in Figure 5; the same data are to be analyzed in detail in this section. Since the colored cells are easily identifiable within whole organ structures, scientists can track the cells and determine their role during processes such as organ regeneration, malignant outgrowth, or immune responses.

To this end, scientists are required to cluster cell types according to some basic color combinations. Due to the variability of the vector insertion, however, single RGB-marked cells express fluorescent proteins at different and very characteristic levels. The underlying principle of additive color mixing, similar to that in computer or TV screens, generates different color combinations that can be used to discriminate individual cell clones. The main difficulty in this kind of data is that the intrinsic variability of the underlying biological mechanisms makes the actual number of distinguishable colors generated by RGB marking in a tissue difficult to predict. In addition, cell intensities for different colors are known to vary depending on the cell area, which is an indicator of cell morphology.

The data set analyzed in this section consists of measurements on colorectal cancer cell lines expressing various quantities of three different fluorescent proteins: Cerulean (blue), Venus (yellow/green), and mCherry (red). The genes coding for the fluorescent proteins were transferred into the cells via lentivirus-mediated transduction at a less than 100% efficiency so that most cells expressed different quantitative combinations of all three fluorescent proteins as described by Weber et al. [24]. The cells were imaged on a high-content imager (Operetta, Perkin Elmer). The final data consisted of fluorescent intensities of red, blue, and green color channels (electromagnetic wavelength in nanometers, nm), morphology parameters including cell areas, and spatial coordinates for 128 cells.

Figure 5 shows the original data and clustering obtained by the LS regression clustering approach defined in (14), using multivariate regression with color intensities as the response vector, with morphological predictor (cell area) being used in (c), and without using any predictors in (d). Clustering methods are relatively robust to the initial random seed (here we used set.seed(111)) in both cases. When the cell area predictor is included, the resulting clustering changes, thus suggesting that the cell morphology information (cell area) plays a role in separating different cells types. In Table 9, we summarize the outcome for this LS regression clustering.

**Figure 6:**
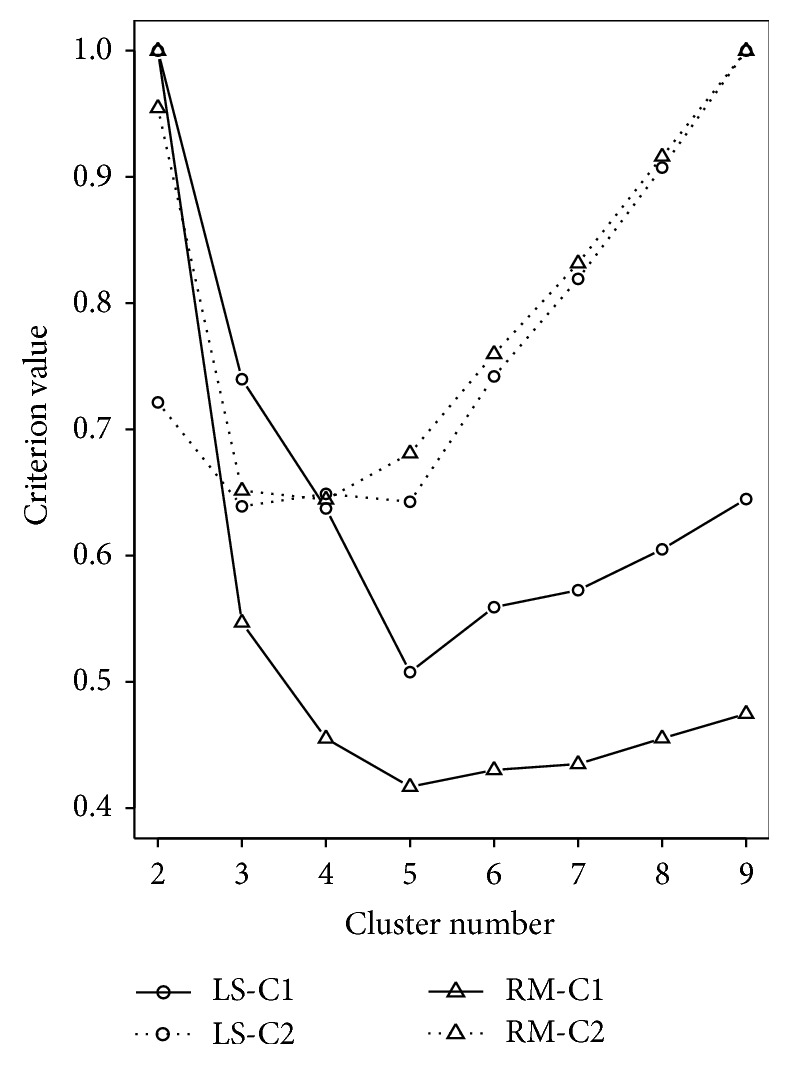
Model selection based on information criterion (8) with *A*_*n*_ equal to log⁡log⁡*n* (C1) and log⁡*n* (C2) for least squares (LS) and robust *M*-estimation (RM) based regression clustering approaches, respectively. All criterion values are scaled between 0 and 1.

To select the optimal number of clusters, we used the information criterion function (8) for LS and (11) for RM, with *q*(*k*) = *k*, where *k* is the unknown number of clusters that we are seeking for.  Figure 6 shows the optimal numbers of clusters using *A*_*n*_ = log⁡log⁡*n* (C1) and *A*_*n*_ = log⁡*n* (C2) for both clustering approaches. Robust clustering is carried out using Huber's discrepancy function (6) with the tuning constant *c* = 1.345 being chosen. The resulting optimal number of clusters based on C1 is 5 by both LS and RM regression clustering criteria, which is compatible with biological considerations.

Finally, we assess the performances of the LS and RM regression clustering and compare them with that of the *k*-means method. The prediction strength (PS) statistic introduced by Tibshirani and Walther [25] is used for the assessment.

For a candidate number of clusters *k* (*k* = 5 in our case), let ${\hat{𝒞}}_{\text{te}}=\left\{{\hat{𝒞}}_{\text{te},1},\ldots ,{\hat{𝒞}}_{\text{te},k}\right\}$ denote the partition of the test set resulting from regression clustering on all the data. Let *n*_1_,…, *n*_*k*_ be the number of observations in these clusters. Let ${\hat{𝒞}}_{\mathrm{tr}}$ be the partition of the test set resulting from regression clustering on the training set. In particular, in the latter case each data point in the test set is clustered using (4) with ${\hat{\beta }}_{i},\;\;i=1,\ldots ,k$, produced by the training set.

Following notations of Tibshirani and Walther [25], denote $D[{\hat{𝒞}}_{\mathrm{tr}},{\hat{𝒞}}_{\text{te}}]$ as the *n* × *n* comembership matrix, with *ii*′th element $D[{\hat{𝒞}}_{\mathrm{tr}},{\hat{𝒞}}_{\text{te}}{]}_{ii^{\prime }}=1$, if a pair of observations *i* and *i*′ that belong to the same cluster in ${\hat{𝒞}}_{\text{te}}$ (i.e., $i\neq i^{\prime }\in {\hat{𝒞}}_{\text{te},j},\;\;j=1,\ldots ,k$) also fall into the same cluster in ${\hat{𝒞}}_{\mathrm{tr}}$, and 0 otherwise. The prediction strength statistic can be written as

(17) $$\begin{array}{c} \text{PS}=\underset{1\leq j\leq k}{\min}\frac{1}{n_{j}\left(n_{j}-1\right)}\underset{i\neq i^{\prime }\in {\hat{C}}_{\text{te},j}}{\sum }D{\left[\;{\hat{C}}_{\mathrm{tr}},{\hat{C}}_{\text{te}}\;\right]}_{ii^{\prime }}. \end{array}$$

Therefore, the prediction strength is the proportion of observation pairs in the worst performing test cluster whose clustering results remain unchanged when clustering them by the training set clustering rule. Clearly, a regression clustering result has higher predictive power if the associated PS is higher.

For our data, we assess the clustering performance by cross-validation using 4 random partitions of our sample. Cross-validated prediction strength values for *k*-means, LS, and RM regression clustering methods are 0.44, 0.80, and 0.66, respectively. This suggests that the LS regression clustering is superior to the *k*-means. Moreover, due to the absence of strong deviations from the multivariate normal model for these data, the out-of-sample prediction strength of the LS regression clustering is larger than that of the robust RM regression clustering approach.

## 7. Conclusion

In this paper, we review the general cluster analysis methods and then focus on regression clustering which uses the model based fixed partition method and clusters the data based on the dependence between the response and explanatory variables. We provide both least squares based and robust *M*-estimation based methods for estimating parameters, partitioning the data, and selecting the optimal number of clusters in regression clustering. Algorithms have been developed to implement these methods. The example and simulation study conclude a satisfactory finite sample performance of the algorithms. Applying our developed method to regression cluster the RGB cells tracking data gives results compatible with biological considerations. It is known that the methods can only provide a local optimization solution and are computing intensive especially when the sample size is large. Currently, we are investigating these issues and expect to provide an improved solution to be reported elsewhere in the near future.

## Figures and Tables

**Table 1:** Confusion matrix between *k*-means clustering and species information.

	* setosa *	* versicolor *	* virginica *
Cluster 1	50	0	0
Cluster 2	0	48	14
Cluster 3	0	2	36

**Table 2:** Confusion matrix between s epal . length ~ sepal.width regression clusters and species information.

	* setosa *	* versicolor *	* virginica *
Cluster 1	50	1	1
Cluster 2	0	35	16
Cluster 3	0	14	33

**Table 3:** Confusion matrix between s epal.length ~ sepal .width + petal.length + petal.width regression clusters and species information.

	* setosa *	* versicolor *	* virginica *
Cluster 1	25	17	14
Cluster 2	12	19	15
Cluster 3	13	14	21

**Table 4:** Shorthand notation for six cases.

N1C2	Case 1	Two regression lines	Normal error
T1C2	Case 2	Two regression lines	*t*(3) error
C1C2	Case 3	Two regression lines	Cauchy(0,1) error
N1C3	Case 4	Three regression lines	Normal error
T1C3	Case 5	Three regression lines	*t*(3) error
C1C3	Case 6	Three regression lines	Cauchy(0, 1) error

**Table 5:** Parameter values used in the simulation study of regression clustering.

Case	*k*_0_	Regression coefficients	Number of observations
1–3	2	$\beta_{01}=\left(\begin{array}{c} 2\\ 8 \end{array}\right)$, $\beta_{02}=\left(\begin{array}{c} 1\\ 5 \end{array}\right)$	*n*_1_ = 70 , *n*_2_ = 50

4–6	3	$\beta_{01}=\left(\begin{array}{c} 18\\ 6 \end{array}\right)$ , $\beta_{02}=\left(\begin{array}{c} 12\\ 8 \end{array}\right)$, $\beta_{03}=\left(\begin{array}{c} 15\\ -2 \end{array}\right)$	*n*_1_ = 35 , *n*_2_ = 35 , *n*_3_ = 50

**Table 6:** Relative frequencies of selecting *k* based on 1000 simulations for Cases  1–3.

*k*_0_ = 2	Case 1 (*N*(0,1) error)		Case 2 (*t*(3) error)		Case 3 (Cauchy(0,1) error)	
	LS-C	RM-C	LS-C	RM-C	LS-C	RM-C
*k* = 1	0.000	0.000	0.001	0.001	0.005	0.006
*k* = 2	0.986	1.00	0.422	0.999	0.292	0.745
*k* = 3	0.014	0.000	0.488	0.000	0.415	0.183
*k* = 4	0.000	0.000	0.087	0.000	0.227	0.055
*k* = 5	0.000	0.000	0.002	0.000	0.061	0.011

**Table 7:** Relative frequencies of selecting *k* based on 1000 simulations for Cases 4–6.

*k*_0_ = 3	Case 4 (*N*(0,1) error)		Case 5 (*t*(3) error)		Case 6 (Cauchy(0,1) error)	
	LS-C	RM-C	LS-C	RM-C	LS-C	RM-C
*k* = 1	0.000	0.000	0.000	0.000	0.000	0.000
*k* = 2	0.000	0.000	0.000	0.002	0.117	0.012
*k* = 3	1.00	1.00	0.791	0.997	0.232	0.611
*k* = 4	0.000	0.000	0.207	0.001	0.566	0.350
*k* = 5	0.000	0.000	0.002	0.000	0.085	0.027

**Table 8:** The estimation of the regression parameters by applying LS-C and RM-C to the data shown in Figures 3 and 4.

*k*_0_	Case	Clusters	**β**_1_	**β**_2_	**β**_3_	**β**_4_
		True	$\left(\begin{array}{c} 2\\ 8 \end{array}\right)$	$\left(\begin{array}{c} 1\\ 5 \end{array}\right)$		
	1	LS-C	$\left(\begin{array}{c} 2.12\\ 8.02 \end{array}\right)$	$\left(\begin{array}{c} 0.76\\ 5.11 \end{array}\right)$		
		RM-C	$\left(\begin{array}{c} 2.11\\ 8.03 \end{array}\right)$	$\left(\begin{array}{c} 0.78\\ 5.10 \end{array}\right)$		
2	2	LS-C	$\left(\begin{array}{c} 1.48\\ 5.56 \end{array}\right)$	$\left(\begin{array}{c} -1.13\\ 5.87 \end{array}\right)$	$\left(\begin{array}{c} 4.46\\ 6.18 \end{array}\right)$	
		RM-C	$\left(\begin{array}{c} 2.21\\ 7.89 \end{array}\right)$	$\left(\begin{array}{c} 0.73\\ 4.89 \end{array}\right)$		
	3	LS-C	$\left(\begin{array}{c} 2.40\\ 6.66 \end{array}\right)$	$\left(\begin{array}{c} -46.33\\ -11.23 \end{array}\right)$		
		RM-C	$\left(\begin{array}{c} 2.29\\ 8.42 \end{array}\right)$	$\left(\begin{array}{c} 0.59\\ 5.17 \end{array}\right)$		

		True	$\left(\begin{array}{c} 18\\ 6 \end{array}\right)$	$\left(\begin{array}{c} 12\\ 8 \end{array}\right)$	$\left(\begin{array}{c} 15\\ -2 \end{array}\right)$	
	4	LS-C	$\left(\begin{array}{c} 18.05\\ 6.06 \end{array}\right)$	$\left(\begin{array}{c} 11.97\\ 8.02 \end{array}\right)$	$\left(\begin{array}{c} 14.66\\ -1.85 \end{array}\right)$	
		RM-C	$\left(\begin{array}{c} 18.04\\ 6.07 \end{array}\right)$	$\left(\begin{array}{c} 11.95\\ 8.03 \end{array}\right)$	$\left(\begin{array}{c} 14.66\\ -1.87 \end{array}\right)$	
3	5	LS-C	$\left(\begin{array}{c} 17.74\\ 6.14 \end{array}\right)$	$\left(\begin{array}{c} 12.02\\ 8.16 \end{array}\right)$	$\left(\begin{array}{c} 10.73\\ -2.87 \end{array}\right)$	$\left(\begin{array}{c} 15.54\\ -1.70 \end{array}\right)$
		RM-C	$\left(\begin{array}{c} 17.88\\ 5.98 \end{array}\right)$	$\left(\begin{array}{c} 12.14\\ 8.14 \end{array}\right)$	$\left(\begin{array}{c} 14.94\\ -1.88 \end{array}\right)$	
	6	LS-C	$\left(\begin{array}{c} 18.23\\ 6.29 \end{array}\right)$	$\left(\begin{array}{c} 12.28\\ 8.27 \end{array}\right)$	$\left(\begin{array}{c} 15.20\\ -2.10 \end{array}\right)$	$\left(\begin{array}{c} 32.17\\ -27.23 \end{array}\right)$
		RM-C	$\left(\begin{array}{c} 18.02\\ 6.26 \end{array}\right)$	$\left(\begin{array}{c} 12.24\\ 7.99 \end{array}\right)$	$\left(\begin{array}{c} 15.19\\ -2.09 \end{array}\right)$	

**Table 9:** Summary statistics based on the 5 clusters obtained from the multivariate LS regression clustering including the cell area covariate: sample means and standard deviations of the 3-dimensional response vector (i.e., RGB intensities on log scale), as well as the number of observations (i.e., cells) in each cluster.

Cluster	Mean (red, green, and blue)	$\hat{SD}$ (red, green, and blue)	Cluster size
1	(4.99, 5.02, 5.78)	(0.10, 0.17, 0.25)	25
2	(5.66, 5.83, 5.78)	(0.23, 0.26, 0.18)	23
3	(5.40, 5.36, 5.57)	(0.12, 0.18, 0.14)	20
4	(4.55, 4.19, 5.52)	(0.28, 0.16, 0.11)	41
5	(6.32, 6.50, 5.92)	(0.22, 0.24, 0.18)	19
